# Supplementary material for: A Novel Module Promotes Horizontal Gene Transfer in Azorhizobium caulinodans ORS571
Source: Genes (Basel). 2022 Oct 19;13(10):1895. doi: 10.3390/genes13101895 (PMC9601964; doi:10.3390/genes13101895)
Supplement: Supplementary file 1 [file genes-13-01895-s001.zip › genes-1934010-supplementary.pdf]

Supporting Information

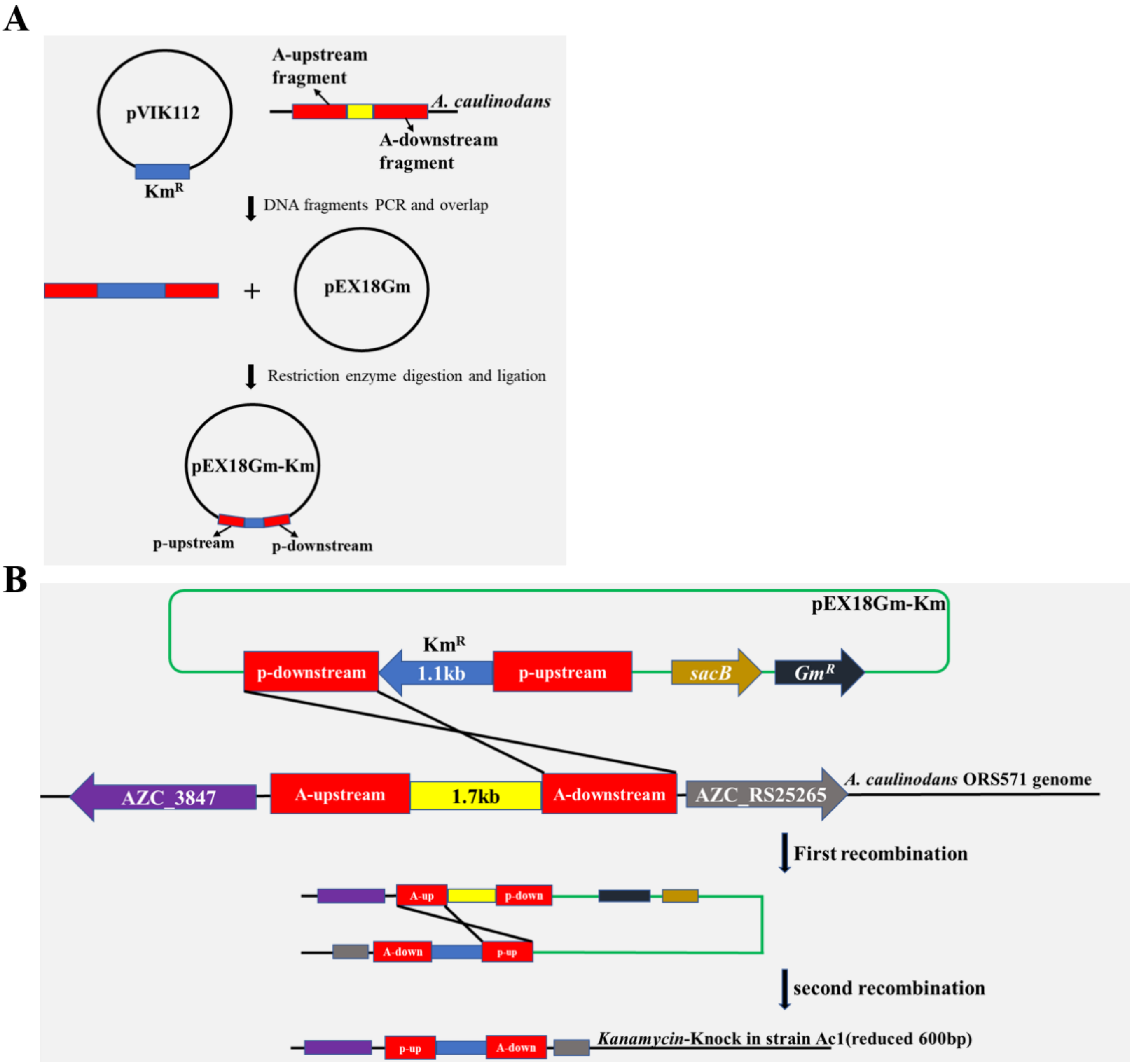

Figure S1. Schematic for the construction of gene insertion and knockout. (**A**) Construction of recombinant plasmid pEX18Gm containing homologous fragments. Using pEX18Gm-Km as an illustration, the fragments were overlapped with each other and inserted into multiple cloning site. (**B**) Homologous recombination of recombinant plasmid in recipient cell. The software SnapGene was used to create recombinant plasmid flowchart.

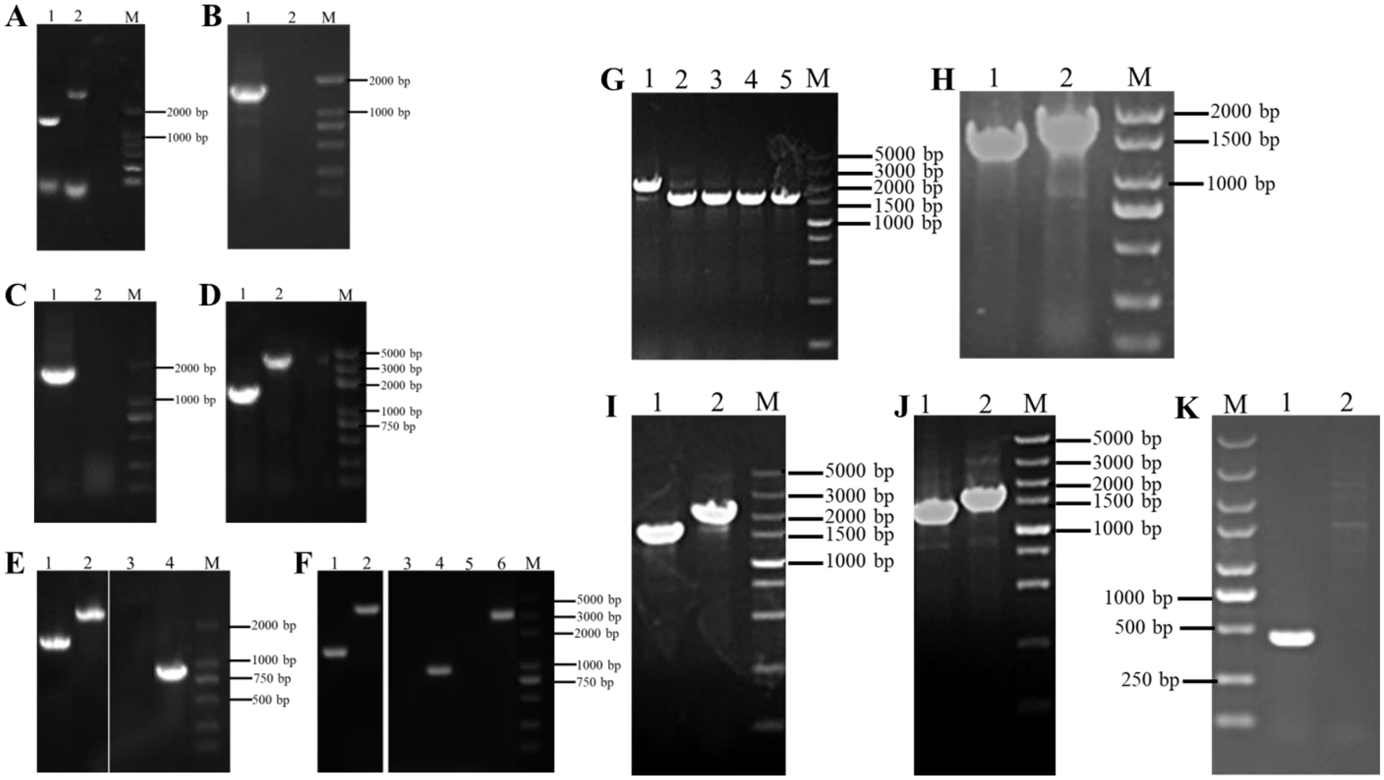

Figure S2. Confirmation of mutant strains. (**A**) Confirmation of *rgf1* in-frame deletion in Azc1 by PCR. Lane 1: Only 1500 bp outside sequence of *rgf1* could be amplified from Δ*rgf1* through primers V*rgf1*-1 and V*rgf1*-4. Line 2: The outside sequence and *rgf1* could be amplified together using same primers in Azc1. (**B**) Confirmation of Δ*rgf2*. Lane 1: Flanking sequence in Δ*rgf2*. Line 2: The complete 4.5 kb fragment of *rgf2* could not be amplified within the 30s PCR extension time using same primers in Azc1. (**C**) Confirmation of Δ*rgf3*. Lane 1: Flanking sequence of *rgf3* in Δ*rgf3*. Line 2: The 9.2 kb of *rgf3* could not be amplified in Azc1. (**D**) Confirmation of Δ*rgf4*. Lane 1: Flanking sequence of *rgf4* in Δ*rgf4*. Line 2: The 2.3 kb of *rgf4* in Azc1. (**E**) Confirmation of Δ*rgf12*. Lane 1: Flanking sequence of *rgf1* in Δ*rgf12*. Line 2: The 1.9 kb *rgf1* in Azc1. Line 3: The internal sequence of *rgf2* was amplified through primers V*rgf2*-inside-1 and V*rgf2*-inside-2 in Δ*rgf12*. Line 4: The internal sequence of *rgf2* in Azc1. (**F**) Confirmation of Δ*rgf123*. Line 1: Flanking sequence of *rgf1* in the mutant. Line 2: 1.9 kb *rgf1* in Azc1. Line 3: The internal sequence of *rgf2* in the mutant. Line 4: The internal sequence of *rgf2* in Azc1. Line 5: The internal sequence of *rgf3*. Line 6: The internal sequence of *rgf3* in Azc1. (**G**) Confirmation of Δ*rihR*. Line 1: The complete fragment of *rihR* in Azc1. Line 2-5: Flanking sequence of *rihR* in Δ*rihR*. (**H**) Confirmation of Δ*rihR**. Line 1: Flanking sequence of *rihR* in Δ*rihR**. Line 2: The fragment of *rihR* in Azc2. (**I**) Confirmation of Δ*rihF1*. Line 1: Flanking sequence of *rihF1* in the mutant. Line 2: The fragment of *rihF1* in Azc1. (**J**) Confirmation of Δ*rihF2*. Line 1: Flanking sequence of *rihF2* in the mutant. Line 2: The fragment of *rihF2* in Azc1. (**K**) Confirmation of Δ*ahaR*. Line 1: The internal sequence of *ahaR* in Azc1. Line 2: The internal sequence of *ahaR* in Δ*ahaR*. M: marker.

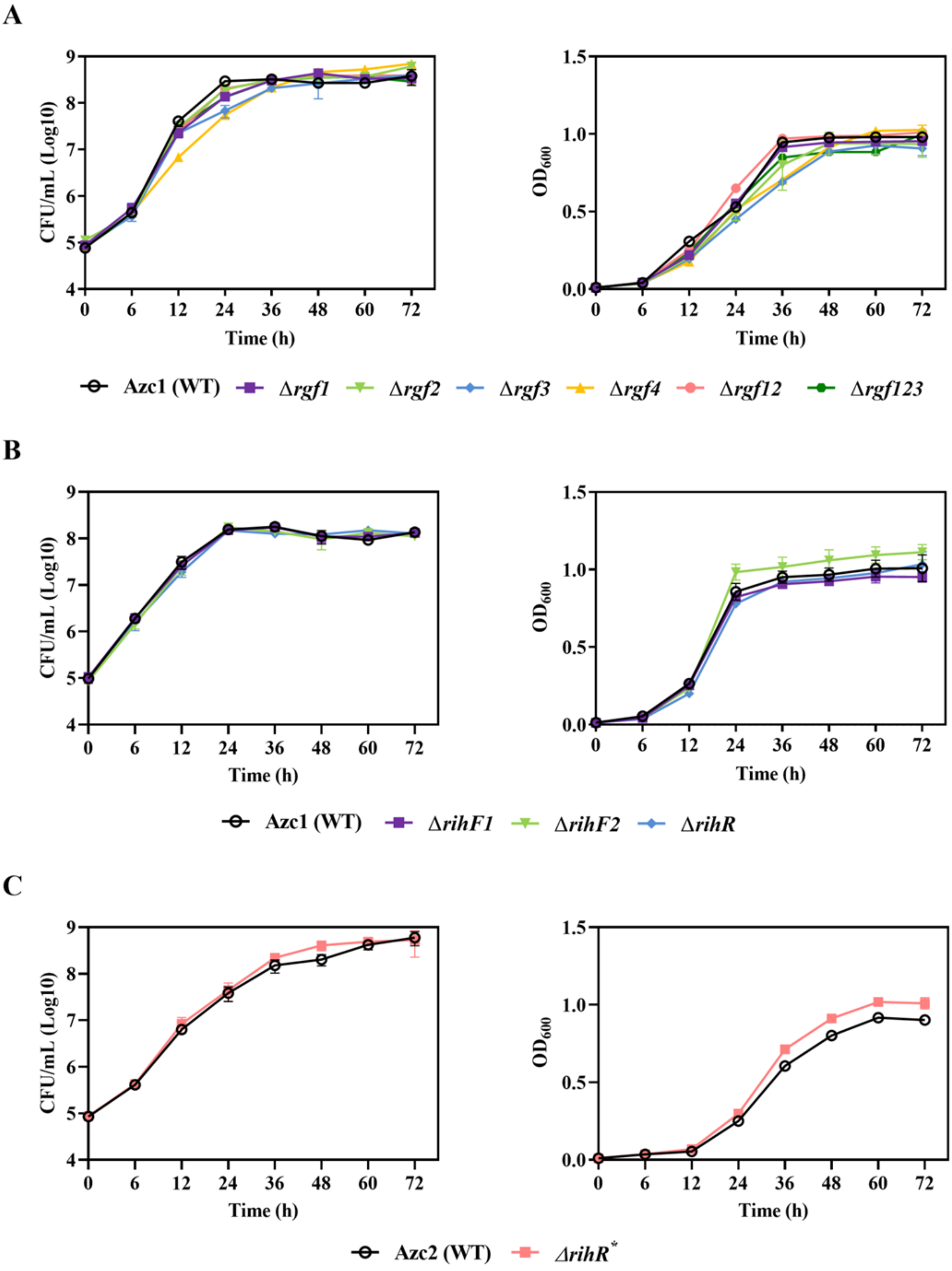

Figure S3. Bacterial growth curves of *A. caulinodans* and its derivative strains. (**A**) The growth curves of Azc1 and its derivatives. (**B**) The growth curves of Azc1 and its derivatives. (**C**) The growth curves of Azc2 and its derivative. Strains were grown in TY medium at 28°C, and the cell density was detected at different time points. The experiment was repeated at least three times.

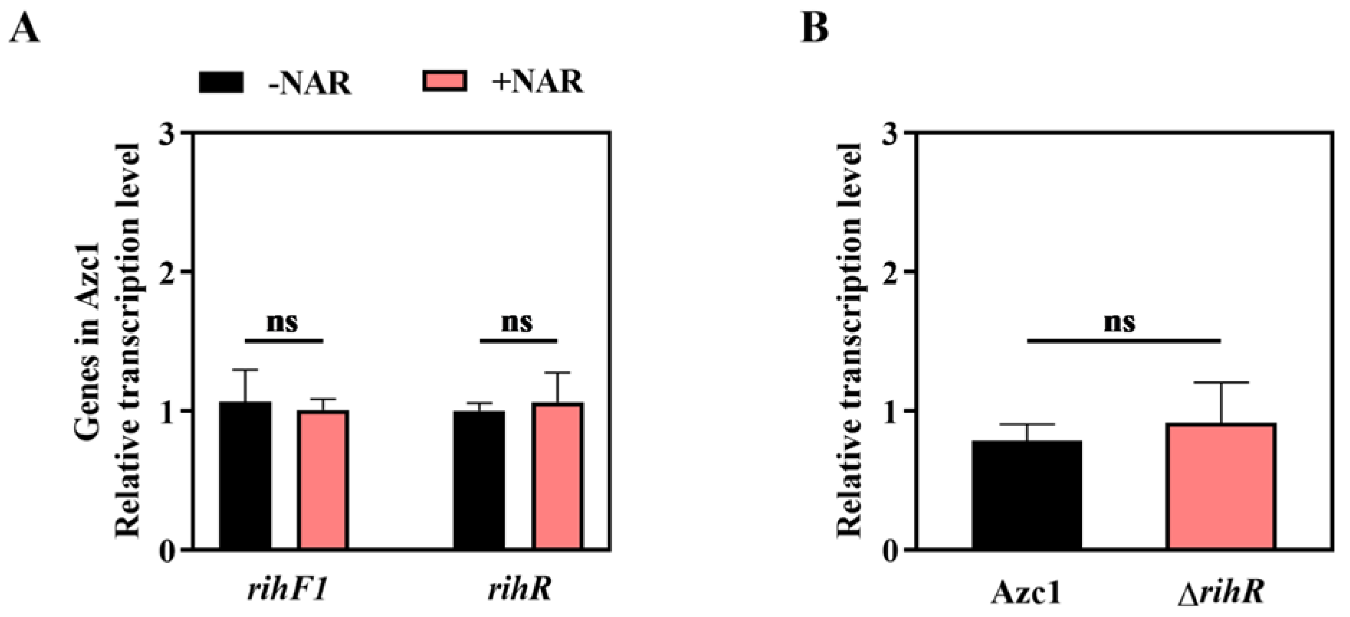

Figure S4. qRT-PCR analysis of the transcriptional levels of the *rihF1*, *rihR* and *ahaR*. (**A**) TranScheme 1. and *rihR* in strain Azc1.TY medium was supplemented with 20 μM NAR or not. (B) Transcriptional levels of *ahaR* in strains Azc1 and Δ*rihR*. The mid-log growing cells were lysed to extract the RNA of qRT-PCR analysis. 16S rRNA was selected as a reference. Data are mean and SD of three independent experiments. ns: no significant difference.

Table S1. Strains and plasmids used in this study.

Strains or Plasmids	Relevant characteristics	Source
A. caulinodans strains		
WT	*Azorhizobium caulinodans* ORS571, Azc0 wild-type, Amp^R	[1]
Azc1	Derivative of WT, Km resistant gene-knock in, Amp^R, Km^R	[2]
Azc2	Derivative of WT, Tet resistant gene-knock in, Amp^R, Tet^R	This study
$Azc1^{Vec}$	Derivative of Azc1 carrying on vector pSRKGm, Amp^R, Km^R, Gm^R	This study
Δ*ahaR*	Derivative of Azc1 carrying on AZC_3869 in-frame deletion, Amp^R, Km^R	This study
Δ*rgf1*	Derivative of Azc1 carrying on 1.9 kb fragment in-frame deletion (AZC_3836 to AZC_3837), Amp^R, Km^R	This study
Δ*rgf2*	Derivative of Azc1 carrying on 4.5 kb fragment in-frame deletion (AZC_3794 to AZC_3800), Amp^R, Km^R	This study
Δ*rgf3*	Derivative of Azc1 carrying on 9.2 kb fragment in-frame deletion (AZC_3841 to AZC_3847), Amp^R, Km^R	This study
Δ*rgf4*	Derivative of Azc1 carrying on 2.3 kb fragment in-frame deletion (AZC_3878 to AZC_3880), Amp^R, Km^R	This study
Δ*rgf12*	Derivative of Azc1 carrying on 6.4 kb fragment in-frame deletion, Amp^R, Km^R	This study
Δ*rgf123*	Derivative of Azc1 carrying on 15.6 kb fragment in-frame deletion, Amp^R, Km^R	This study
Δ*rihR**	Derivative of Azc2, AZC_3881 in-frame deletion mutant, Amp^R, Tet^R	This study
Δ*rihR*	Derivative of Azc1, AZC_3881 in-frame deletion mutant, Amp^R, Km^R	This study
Δ*rihF1*	Derivative of Azc1, AZC_3879 and AZC_RS26200 double in-frame deletion mutant, Amp^R, Km^R	This study
Δ*rihF2*	Derivative of Azc1, AZC_3880 in-frame deletion mutant, Amp^R, Km^R	This study
Δ*rihR*^{Vec}	Derivative of Δ*rihR* carrying on vector pSRKGm, Amp^R, Km^R, Gm^R	This study
Δ*rihR*^{C}	Derivative of Δ*rihR* harboring expression plasmid pSRKGm-*rihR*, Amp^R, Km^R, Gm^R	This study
Δ*rihF1*^{Vec}	Derivative of Δ*rihF1* carrying on vector pSRKGm, Amp^R, Km^R, Gm^R	This study
Δ*rihF1*^{C}	Derivative of Δ*rihF1* harboring expression plasmid pSRKGm-*rihF1*, Amp^R, Km^R, Gm^R	This study
Δ*rihF1* (*rihF1a*)	Derivative of Δ*rihF1* harboring expression plasmid pSRKGm-*rihF1a*, Amp^R, Km^R, Gm^R	This study
Δ*rihF1* (*rihF1b*)	Derivative of Δ*rihF1* harboring expression plasmid pSRKGm-*rihF1b*, Amp^R, Km^R, Gm^R	This study
Mesorhizobium huakuii 93	Derivative of Wild type *M. huakuii* 93, spontaneous Spe^R	[2]
E. coli strains		
DH5*α* λpir	Host for cloning	[3]
SM10 λpir	Host for conjugation	[4]
BL21 (DE3)	Host for protein expression	[3]
XL1-Blue MRF′ Kan	Host for bacterial one-hybrid assay	[5]
Plasmids		

pEX18Gm	Suicide cloning vector, Gm^R	[6]
pSRKGm	Gene expression vector for genetic complementation, Gm^R	Lab strain
pVIK112	Transcriptional fusion vector, Km^R	[7]
pRA302	Translational fusion vector, Spe^R	[8]
pTRG	The plasmid used for protein expression in bacterial one-hybridization assay, Tet^R	[9]
pBXcmT	The plasmid used for DNA cloning in bacterial one-hybridization assay, Chl^R	[9]
pET-28a	Protein expression vector, Km^R	[10]
pET28a-*rihR*	Vector for the expression of RihR protein	This study

Table S2. PCR primers used in this study.

Primer	Sequence (5′-3′)[a]	Restriction sites
For deletion and insertion		
Azc2-1	AACTGCAGGCGTATAAATATTCAGCAGC	*Pst*I
Azc2-2	TGTTGGTCGGCGGCCGCGCGGAAACATCTGCCTCACT	
Azc2-3	ATAAGAATGCGGCCGCTATAAAAATAGGCGTATCAC	
Azc2-4	ATAAGAATGCGGCCGCTTGGGCGCATTTGCGCATTC	
Azc2-5	TGTTTCCGCGCGGCCGCCGACCAACATCACTCCTGTC	
Azc2-6	CGGAGCTCTCTTTGCGCCGAGCACCTAC	*Sac*I
Δ*ahaR*-1	GGGGTACCAGCTCGCCATGGATGATCAG	*Kpn*I
Δ*ahaR*-2	AAATGAGAAGCGAGCGTCGAATGTCGTCAC	
Δ*ahaR*-3	TCGACGCTCGCTTCTCATTTGCCGGCATGA	
Δ*ahaR*-4	TGCTCTAGACATCGTGGGCATAGCCGTCG	*Xba*I
Δ*rgf1*-1	CGGAATTCACGCGATGGGTCTTGGAGAG	*Eco*RI
Δ*rgf1*-2	AACTTGGACCGTCTACAATCGAGCCCTATG	
Δ*rgf1*-3	GATTGTAGACGGTCCACGTTCCACGGATTG	
Δ*rgf1*-4	GCTCTAGAATGTACTGGGAGGCGCCATC	*Xba*I
Δ*rgf2*-1	CGGAATTCATCGCTGGTTGCGAGCATAC	*Eco*RI
Δ*rgf2*-2	ATCAACCGGTATCCATTGCCTCAGGATGAC	
Δ*rgf2*-3	GGCAATGGATACCGGTTGATCCAGATGTTG	
Δ*rgf2*-4	GCTCTAGAGCCCTGATGCTCTGCTTGAC	*Xba*I
Δ*rgf3*-1	CGGAATTCTCCACGTTCCACGGATTG	*Eco*RI
Δ*rgf3*-2	CACCTATACCCCGGTCTATCGCCTCAG	
Δ*rgf3*-3	GATAGACCGGGGTATAGGTGCGCGTG	
Δ*rgf3*-4	GCTCTAGATGCGCAAATTTCCGAGC	*Xba*I
Δ*rgf4*-1	CGGAATTCATCCATTGGAAAGGCGGA	*Eco*RI
Δ*rgf4*-2	CATATGACCGGTTCGATCCGTGCACC	
Δ*rgf4*-3	CGGATCGAACCGGTCATATGACACCGC	
Δ*rgf4*-4	CCAAGCTTAGCTGCTCATTCCATCTC	*Hind*III
Δ*rihR**-1	GCTCTAGAGCCTGACGATCGCCTGGTATTG	*Xba*I
Δ*rihR**-2	CTCGGCTGGGCAGACGGATCGCAATTTACG	
Δ*rihR**-3	GATCCGTCTGCCCAGCCGAGATGGAATGAG	
Δ*rihR**-4	CGGAATTCGCCGGTAAGACTTCGAAAGC	*Eco*RI
Δ*rihR*-1	GCTCTAGAGCCTGACGATCGCCTGGTATTG	*Xba*I
Δ*rihR*-2	CTCGGCTGGGCAGACGGATCGCAATTTACG	
Δ*rihR*-3	GATCCGTCTGCCCAGCCGAGATGGAATGAG	
Δ*rihR*-4	CGGAATTCGCCGGTAAGACTTCGAAAGC	*Eco*RI
Δ*rihF1*-1	GCTCTAGACGCAGCACCTTACGGAACAG	*Xba*I
Δ*rihF1*-2	GTTTGGTCGCACGACCGTAGCTTCGTCTTC	
Δ*rihF1*-3	CTACGGTCGTGCGACCAAACGATGGATTGC	
Δ*rihF1*-4	CGGAATTCCGCCCTCCGAGATTATCAAC	*Eco*RI
Δ*rihF2*-1	GCTCTAGAAACAGGGCTACGGCGATGTC	*Xba*I
Δ*rihF2*-2	ACCGGCTACGAGATGCAGTCAGGGCATCAC	
Δ*rihF2*-3	GACTGCATCTCGTAGCCGGTGAGTTGGAGG	
Δ*rihF2*-4	CGGAATTCTCAGTCGATCGTCGCAACAC	*Eco*RI
For verification of mutants		
V*rgf1*-1	ACGCGATGGGTCTTGGAGAG	
V*rgf1*-4	ATGTACTGGGAGGCGCCATC	
V*rgf2*-1	ATCGCTGGTTGCGAGCATAC	

V*rgf2*-4	GCCCTGATGCTCTGCTTGAC	
V*rgf3*-1	TCCACGTTCCACGGATTG	
V*rgf3*-4	TGCGCAAATTTCCGAGC	
V*rgf4*-1	ATCCATTGGAAAGGCGGA	
V*rgf4*-4	AGCTGCTCATTCCATCTC	
V*rgf2*-inside-1	CGGCAAACTGCTCGAAGCTAGTGAGCAT	
V*rgf2*-inside-2	GACGGAATCGGCGCGGCCA	
V*rgf3*-inside-1	GGAAGGACGATGGCCGTGGA	
V*rgf3*-inside-2	TATGCCATCCGTCGCATGCT	
V*rihR*-1	GCCTGACGATCGCCTGGTATTG	
V*rihR*-4	GCCGGTAAGACTTCGAAAGC	
V*rihR**-1	GCCTGACGATCGCCTGGTATTG	
V*rihR**-4	GCCGGTAAGACTTCGAAAGC	
V*rihF1*-1	CGCAGCACCTTACGGAACAG	
V*rihF1*-4	CGCCCTCCGAGATTATCAAC	
V*rihF2*-1	AACAGGGCTACGGCGATGTC	
V*rihF2*-4	TCAGTCGATCGTCGCAACAC	
V*ahaR*-inside-1	GCAAATCACTGCCGCGTACT	
V*ahaR*-inside-2	CACACGCCTTTTATCGCGAT	
For gene complementary		
pSRKGm-*rihR*-F	GCTCTAGAAGGGCGTAAATTGCGATCCG	*Xba*I
pSRKGm-*rihR*-R	GGGGTACCACGACGAGGACGGCAATACC	*Kpn*I
pSRKGm-*rihF1*-F	GCTCTAGACCCTTAGGCAAAGCAATTCC	*Xba*I
pSRKGm-*rihF1*-R	GGGGTACCGCGGAGGATAGGGAAGTTGG	*Kpn*I
pSRKGm-*rihF1a*-F	GCTCTAGAATGGCAGCCGTCATTCCGAT	*Xba*I
pSRKGm-*rihF1a*-R	GGGGTACCCTACGGTCGTCGACGATGAC	*Kpn*I
pSRKGm-*rihF1b*-F	GCTCTAGAATGAAGCCGGACACGTCGCG	*Xba*I
pSRKGm-*rihF1b*-R	GGGGTACCTCACCGATGGAGGCGTGAGC	*Kpn*I
For transcriptional fusion		
pVIK112-*rihF1*-F	CCCCCGGGAGGGAGGTCGACGGTATTTC	*Sma*I
pVIK112-*rihF1*-R	GGGGTACCCTTTGACGAGGCCGATGACC	*Kpn*I
For translational fusion		
pRA302-*rihR*-F	GGTACCCGGGGATCCTCTAGAGCTGTCAAAGATATTACGCGCA	*Xba*I
pRA302-*rihR*-R	AAAACGACGGGATCGAAGCTTCGCATATCCATGCGCAGAC	*Hind*III
pRA302-*intC*-F	CCGGAATTCTGCGCCGCAAAGACCTGGTG	*Eco*RI
pRA302-*intC*-R	TGCTCTAGACGGTACTGCACGATGAAACT	*Xba*I
pRA302-*ahaR*-F	TGCTCTAGATCAGAAGATCGACCCGCTTG	*Xba*I
pRA302-*ahaR*-R	CCCAAGCTTCGGAAAGCGGCCACATGACT	*Hind*III
For protein expression		
RihR-pET28a-F	CGGAATTCATGGATATGCGCAAGCTGGT	*Eco*RI
RihR-pET28a-R	CCAAGCTTTCAGTCCTTGCCGTCAGGCC	*Hind*III
For EMSA		
P_{intC}-F	ACATCTGGCTCTGCCGAACC	
P_{intC}-R	TCCAAACGGCGCTTTGTGAG	
$P_{control}$-F	CGGAATTCATCGCGGTGGTATTTTTGCG	
$P_{control}$-R	GCTCTAGATGACTGAAACGCATCCTCTC	
For bacterial one-hybrid assay		

pTRG-*rihR*-F	CGGGATCCATGGATATGCGCAAGCTGGT	*BamH*I
pTRG-*rihR*-R	CCGCTCGAGTCAGTCCTTGCCGTCAGGCC	*Xho*I
pBXcmT-*intC*-F	CGGAATTCACATCTGGCTCTGCCGAACC	*EcoR*I
pBXcmT-*intC*-R	GCTCTAGATCCAAACGGCGCTTTGTGAG	*Xba*I
For qRT-PCR		
q*rihF1*-F	GAACCACGCCAGCTATCG	
q*rihF1*-R	AAGTTTTCCAGGGTTCAGAAG	
q*rihR*-F	TTCAGCCAGCAGTACATC	
q*rihR*-R	TGTGCCAGTTCATAGAGC	
q*ahaR*-F	GTACCACCAAAGCACCGACT	
q*ahaR*-R	GCTAGGTCGCGTTTGTCTTC	

[a]The underline sequence is the restriction site of indicated enzymes.

References

1. Goethals, K., M. Van Montagu, and M. Holsters, *Conserved motifs in a divergent nod box of Azorhizobium caulinodans ORS571 reveal a common structure in promoters regulated by LysR-type proteins.* Proc Natl Acad Sci U S A, 1992. **89**(5): p. 1646-50.
2. Ling, J., et al., *Plant nodulation inducers enhance horizontal gene transfer of Azorhizobium caulinodans symbiosis island.* Proc Natl Acad Sci U S A, 2016. **113**(48): p. 13875-13880.
3. Chart, H., et al., *An investigation into the pathogenic properties of Escherichia coli strains BLR, BL21, DH5alpha and EQ1.* J Appl Microbiol, 2000. **89**(6): p. 1048-58.
4. Ferrières, L., et al., *Silent mischief: bacteriophage Mu insertions contaminate products of Escherichia coli random mutagenesis performed using suicidal transposon delivery plasmids mobilized by broad-host-range RP4 conjugative machinery.* J Bacteriol, 2010. **192**(24): p. 6418-27.
5. Xu, K., et al., *Coordinated control of the type IV pili and c-di-GMP-dependent antifungal antibiotic production in Lysobacter by the response regulator PilR.* Mol Plant Pathol, 2021. **22**(5): p. 602-617.
6. Hoang, T.T., et al., *A broad-host-range Flp-FRT recombination system for site-specific excision of chromosomally-located DNA sequences: application for isolation of unmarked Pseudomonas aeruginosa mutants.* Gene, 1998. **212**(1): p. 77-86.
7. Kalogeraki, V.S. and S.C. Winans, *Suicide plasmids containing promoterless reporter genes can simultaneously disrupt and create fusions to target genes of diverse bacteria.* Gene, 1997. **188**(1): p. 69-75.
8. Jiang, G., et al., *Alkyl hydroperoxide reductase is important for oxidative stress resistance and symbiosis in Azorhizobium caulinodans.* FEMS Microbiol Lett, 2019. **366**(3).
9. Guo, M., et al., *Dissecting transcription regulatory pathways through a new bacterial one-hybrid reporter system.* Genome Res, 2009. **19**(7): p. 1301-8.
10. Guan, G., et al., *Multiple sequence elements are involved in the transcriptional regulation of the human squalene synthase gene.* J Biol Chem, 1997. **272**(15): p. 10295-302.
